# Tixagevimab and Cilgavimab Administration for Hemodialysis Patients at Community-Based Dialysis Centers in Singapore as Pre-Exposure Prophylaxis for SARS-CoV-2 Infection

**DOI:** 10.7759/cureus.41297

**Published:** 2023-07-03

**Authors:** Behram A Khan, Marlyn Pagsinohin, Lucy M Lu, Pauline Tan, Rachel Teo

**Affiliations:** 1 Department of Medicine, National University of Singapore, Singapore, SGP; 2 Department of Nursing, The National Kidney Foundation Singapore, Singapore, SGP; 3 Department of Medicine, Duke-National University of Singapore, Singapore, SGP

**Keywords:** community-based, dialysis center, immunocompromised status, sars-cov-2, covid-19, pre-exposure prophylaxis (prep), evusheld, hd ( hemodialysis ), cilgavimab, tixagevimab

## Abstract

Introduction: Hemodialysis patients are deemed to be immunosuppressed and may not be able to mount an adequate response to vaccination against the SARS-CoV-2 virus. Due to the higher morbidity and mortality in this vulnerable group, pre-exposure prophylaxis with monoclonal antibodies was introduced as an additional measure for protection in selected community-based hemodialysis patients in Singapore. Tixagevimab and cilgavimab, available as Evusheld, were used for this purpose.

Methods: A government-sponsored clinical administration program with the provision of 200 doses of Evusheld at no cost to the patients was implemented. Patient selection criteria to further risk-stratify this vulnerable hemodialysis patient cohort was developed and 200 patients were finally selected. Evusheld administration was done over a period of two months, as two consecutive injections were given at two separate intramuscular sites, which constituted one administration. Data were collected as part of a retrospective clinical audit, as part of a routine quality monitoring process for this patient care program. Real-world evidence was generated to assess the impact on mortality, hospitalization rate, reason for hospitalization, and any associated morbidity.

Results: No adverse events from the Evusheld administration were noted. All recipients had received COVID-19 vaccinations prior to Tixa-Cilga, with a range of one to five doses. A total of 198 (99%) completed two doses and 189 (95%) completed three doses, out of which, 14 (7%) patients contracted COVID-19 infection over three months. The overall hospitalization rate was 2% (four out of 200 patients). Severe illness that required intensive care unit stay was therefore seen in only 2 (1%) out of 200 patients. None of the infected patients died.

Discussion: A significant reduction in severity of illness, hospitalization rate, and mortality was found with pre-exposure prophylaxis with tixagevimab and cilgavimab, in this real-world experience from Singapore. Evusheld administration reduced the hospitalization rate from 42.5% to 2%, which is a reduction of 95.3% (p<0.0001). Symptoms in infected patients were mild, with only 1% being admitted to the intensive care unit. The mortality rate from COVID-19 infection was reduced from 2.5% to 0% with Evusheld.

Conclusion: Mass administration of prophylactic treatments for vulnerable populations can be challenging in community-based settings and the successful implementation of such a program has been described. The findings can have health policy implications for the protection of such immunocompromised patients in the future. The combination of tixagevimab and cilgavimab, available as Evusheld in Singapore, was safe to use in hemodialysis patients, with no adverse events noted. There was a significant reduction in hospitalization rates and intensive care unit admissions with a zero-mortality rate due to COVID-19 infection, after pre-exposure prophylaxis.

## Introduction

The coronavirus disease of 2019 (COVID-19) resulted in a global pandemic, which affected millions of people globally, with high mortality rates in vulnerable populations including end-stage kidney disease patients on dialysis [1]. Tixagevimab and cilgavimab (Tixa-Cilga), available under the brand name of Evusheld in Singapore, is a combination of these two human monoclonal antibodies, targeted against the surface spike protein of SARS-CoV-2 virus [2]. Tixa-Cilga was granted an interim authorization under the Pandemic Special Access Route on 1 August 2022 by the Health Science Authority of Singapore [3]. Under this authorization, it could be used for the prevention of COVID-19 in adults who have not had a known recent exposure to an individual with COVID-19 infection (pre-exposure prophylaxis) and are unlikely to mount an adequate immune response to COVID‐19 vaccination due to their immunocompromised state from a medical condition or receipt of immunosuppressive treatments. Furthermore, it could also be used for whom COVID-19 vaccination is not recommended. 

In June 2022, The National Kidney Foundation Singapore (NKF) was approached to offer Tixa-Cilga as pre-exposure prophylaxis for the prevention of COVID-19 infection in hemodialysis (HD) patients, who are immunocompromised with an increased risk of serious and life-threatening complications from such infections [3,4]. We, therefore, conducted a retrospective clinical audit to assess for any changes in morbidity, mortality, and hospitalization in these 200 patients on HD, with a standard dose of 600 mg (300 mg of tixagevimab and cilgavimab each). The objective of the study is to share real-world evidence of the learnings from such a patient care program in affording better protection to HD patients against COVID-19 infections.

## Materials and methods

**Figure 1:**
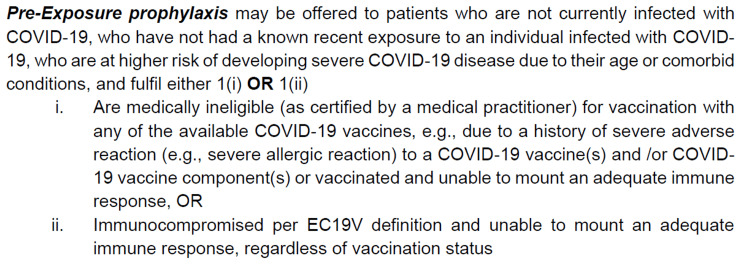
Indication for administering tixagevimab and cilgavimab to hemodialysis patients for pre-exposure prophylaxis against COVID-19 infection. EC19V: Expert Committee on COVID-19 Vaccination of Ministry of Health Singapore. Source: Consent for Evusheld injection. Text derived from the section on “indication” for Evusheld.

The Nursing Infection Control Team (IC Team) and Medical Director (MD), through the guidance of the National Centre for Infectious Diseases (NCID), developed a patient selection criterion, a consent form, and a nursing Quick-Guide, which was approved by The Ministry of Health Singapore. The indication for administering Tixa-Cilga was mentioned in all these documents (Figure 1). 

**Figure 2:**
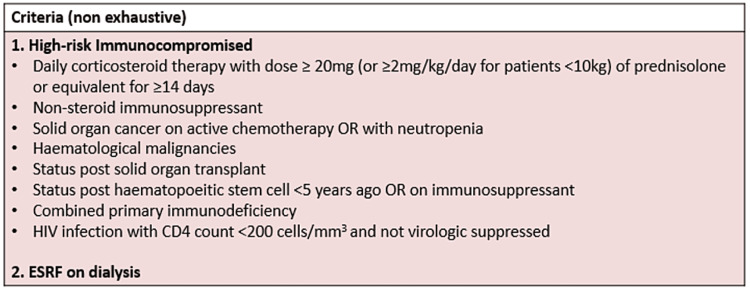
Executive Committee of COVID-19 Vaccination criteria for defining immunocompromised patients. ESRF: end-stage renal failure; HIV: human immunodeficiency virus. Source:  Checklist for Evusheld Injection. Text derived from the section on “definition of moderate to severe immunocompromise”.

Per the "Expert Committee of COVID-19 Vaccination" (EC19V) definition, end-stage renal disease on dialysis was considered immunocompromised [5]. Please see Figure 2, which summarizes the EC19V criteria.

**Figure 3:**
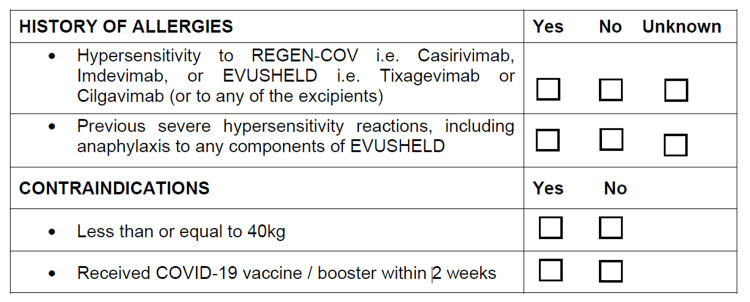
Exclusion criteria for administration of tixagevimab and cilgavimab for pre-exposure prophylaxis against COVID-19 infection. Source: Consent for Evusheld injection. Text derived from the section on “certification of consent” for Evusheld.

The exclusion criteria included the patient’s body weight of less than 40 kg, receiving the COVID-19 vaccine in the past two weeks, and having any type of hypersensitivity reaction, which were detailed in the consent form and nursing guide, as illustrated in Figure 3. 

Nurse managers from 41 dialysis centers were able to shortlist a total of 250 patients initially, based on these selection criteria and those who had preliminarily shown interest in receiving Tixa-Cilga prior to formal consent. Additionally, patients with COVID-19 infection currently or in the last three months were also excluded, as it was felt that these patients would have adequate natural immunity against SARS-CoV-2. These patients were referred to their attending nephrologists for further review and assessment. Finally, 200 patients were selected after this process, with final approval being obtained from the MD for proceeding with the Tixa-Cilga administration. The reason for limiting the patients to 200 was due to the number of cost-free dosages provided by the government for this program.

Tixa-Cilga administration was conducted for two months in October and November 2022. Patients were given the option to receive the IM Tixa-Cilga either pre-dialysis or on non-dialysis days. Only Nurse Managers or Senior Staff Nurses were authorized to administer the drug. Before the injection, the patient’s general condition and vital signs were recorded. Step-By-Step Quick-Guide was available for nurses to consult, with an emergency trolley placed next to the patient to cater for any adverse events. Ice packs for post-administration placement on the injection site were provided for usage. Tixa-Cilga was given intramuscularly at ventro-gluteal and dorso-gluteal sites, as two separate consecutive injections. The patients were monitored closely for 1 hour for any adverse reactions. This included assessment for injection site pain or bleeding, general well-being, and vital signs stability. They were then cleared to either be discharged home or to proceed for HD, depending on the timing of the injection in correlation to their dialysis schedule.

Patient data were analyzed through a retrospective clinical audit to assess the outcomes from this patient care program of pre-exposure Tixa-Cilga administration against COVID-19 infection. The real-world evidence was generated to assess routine quality outcomes such as mortality, hospitalization rate, the reason for hospitalization, and associated morbidity. Recipients’ demographic data (as detailed in the next section) included age, gender, race, COVID-19 vaccination history, immediate adverse event post-injection, COVID-19 infection after Tixa-Cilga administration, level of severity of COVID-19 infection, and any hospitalization that was required. The time interval from the date of administration of Tixa-Cilga to any adverse event was also noted. Data collection was done by all dialysis centers using a secured Microsoft Excel file, which was uploaded into a secured intranet common drive, with the supervision of the IC team to ensure accuracy. The data were then de-identified, anonymized, and presented as aggregated data sets for the clinical audit team to review and analyze.

## Results

A review was conducted after administration to analyze the effect and impact of Tixa-Cilga in these 200 HD patients. No adverse events were recorded after administration, including no prolonged injection-site pain or bleeding, which was assessed up to 1 hour after the injection. Patients who proceeded for their HD after injection completed their HD session without any adverse events.

**Figure 4:**
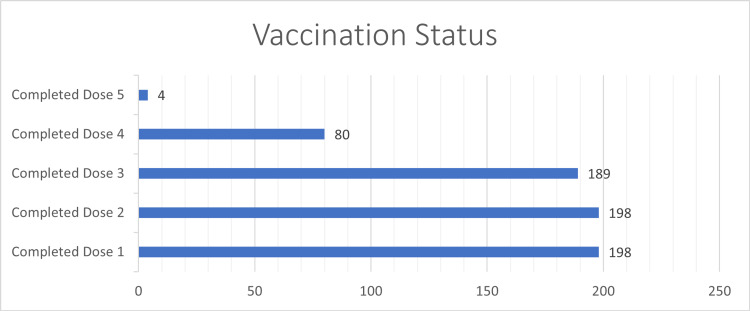
Number of patients receiving COVID-19 vaccination divided into groups based on the number of vaccine doses received

Among all recipients, 137 (68%) were male and 63 (32%) were female. The mean age was 62.9 years, with the youngest aged 38 years and the oldest 86 years. Age-related analysis showed that 11 (6%) belonged to the 20-39 years range, 54 (27%) to 40-59 years range, 74 (37%) to 60-69 years range, and 61 (31%) were above 70 years. The majority were Chinese (61%), followed by Malay (22%), Indian (10%), and other races (8%). All recipients had received COVID-19 vaccinations prior to Tixa-Cilga, with a range of one to five doses. A total of 198 (99%) completed two doses, 189 (95%) completed three doses, 80 (40%) completed four doses, and 4 (2%) completed five doses. Please see Figure 4.

We assessed the effectiveness of Tixa-Cilga in these 200 patients within three months of administration. A total of 14 (7%) patients contracted COVID-19 infection between 4 and 106 days after administration. Among these 14 cases, 10 (71%) had mild symptoms, while 4 (29%) had moderate symptoms requiring hospitalization. Per the ongoing Ministry of Health protocols, these four patients were hospitalized due to moderate symptoms, with an overall hospitalization rate of 2% (four out of 200 patients). Out of these four hospitalized patients, two patients required intensive care unit stay, with both these patients having significant premorbid cardiovascular morbidity. The average length of hospital stay was 8.75 days for the four cases. Severe illness that required intensive care unit stay was therefore seen in only 2 (1%) out of 200 patients. Mortality rates of 0% were noted in the 14 hospitalized cases, as none of the patients died.

As 198 (99%) patients were considered fully vaccinated with two doses and only 14 patients contracted COVID-19, the study size was not adequate to conduct any meaningful statistical analysis of vaccination dosage status and related COVID-19 infection outcomes. 

In comparison, a baseline from April 2020 to August 2022 during the COVID-19 pandemic showed that the same dialysis centers, which consisted of a total of 4425 patients, had 2534 patients with COVID-19 infection. In this baseline group which can be considered as a historic control, hospitalization was required in 1077 patients, which translates to a 42.5% hospitalization rate. A total of 64 patients died in this baseline period due to COVID-19, showing a mortality rate of 2.6% in these dialysis centers due to the pandemic. 

Of note, 98.6% of this historic control group had completed one dose of COVID-19 vaccination and 97.9% had completed two doses. This is in comparison to 99% of the Tixa-Cilga-receiving group having completed two doses of vaccination. There was no statistically significant difference between the fully vaccinated status (defined as two vaccination doses) of the group that received Tixa-Cilga and the ones that did not receive it (p<0.167).

## Discussion

This real-world experience in community-based dialysis centers in Singapore shows a successful clinical program of administering pre-prophylaxis Tixa-Cilga (Evusheld) to HD patients. It is always a challenge to implement community-based mass administration programs for risk reduction of morbidity and mortality in vulnerable population cohorts [6-8]. HD patients are immunocompromised and are at higher risk of morbidity and mortality due to COVID-19 infection, with 200 of such vulnerable patients chosen for pre-exposure prophylaxis against COVID-19 infection [9-11]. This strategy was to optimize the usage of Tixa-Cilga in a cost-effective manner and to exhibit prudent utilization to the health authorities. This had further positive feedback from a health policy planning and implementation perspective, where organizations can implement effective clinical governance to manage such programs, which have future roll-out implications on a national level. 

There was a concern among the nursing team that administering two concurrent injections as part of a single administration of Tixa-Cilga may lead to problems with patient tolerability due to pain or hematoma formation, as routine vaccination against COVID-19 consists of only one injection at a time [12]. However, it was well tolerated and there were no adverse events noted at the injection site or in general in the patients. There was also concern about the short- to medium-term cardiac complications that were reported in the limited literature available with Tixa-Cilga usage [13]. We did not see any vital sign abnormalities, symptoms, or signs suggesting cardiac problems after the administration, as directly documented on a short-term basis as part of the 1 hour after injection administration and subsequent successful completion of the next HD session. After three months of successful completion of the program at these selected dialysis centers, no adverse incident reports were noted suggesting unexplained cardiac or other episodes that may point toward adverse events linked to Tixa-Cilga administration in these patients. These observations were tracked for over three months as part of the clinical audit.

Both genders were included in the study, with three main ethnicities usually identified in Singaporean communities. Inclusion was also noted over a wide age range of treatment participants. Although one indication of Tixa-Cilga administration in high-risk populations would include those who have not had contraindications to COVID-19-related vaccinations, we found that the 200 patients were all immunized. A vast majority comprising 99% of the patients had received at least two doses of vaccinations, showing that the group was adequately vaccinated. This was similar to the baseline comprising all HD patients in the dialysis centers under study (historical control), which showed 97.9% having received two doses. Therefore, the higher COVID-19 infection, hospitalization, and mortality rate cannot be accounted for by any significant difference in the vaccination status in the group receiving Tixa-Cilga compared to historical controls at baseline (p<0.167).

Tixa-Cilga administration reduced the hospitalization rate from 42.5% to 2%, which is a reduction of 95.3% (p<0.0001). This was a significant achievement with public health implications on a national level, as bed availability in the hospitals was severely constrained during peaks of infectivity rates in the COVID-10 pandemic. Furthermore, intensive care unit bed utilization was very low at 1% only. The 7% COVID-19 positivity rate that was seen with those having received Tixa-Cilga had mild symptoms that did not require hospitalization and led to home-based recovery.

The mortality rate was reduced from 2.5% to 0% with no deaths noted in those who received Tixa-Cilga. Although the sample size of 200 patients is not large, this program effectively reduced the mortality rate in this vulnerable HD population to below what was observed in the general population. This result has implications to instruct public health policy regarding the effective utilization of human monoclonal antibodies to counter the morbidity and mortality related to COVID-19 [14]. Public perception of adopting such measures beyond vaccination can be positively influenced by real-world experience sharing of such programs [15]. It also makes an evidence-based case to further the research and development of such therapeutics to effectively protect high-risk populations in the future with the emergence of SARS-CoV-2 variant strains [16,17]. 

The limitation of this study is that it is a real-world experience of a clinical audit and lacks randomization and blinding. This may have contributed to a degree of bias in case selection. Results were limited to community-based HD centers under study in Singapore and may not be generalized to all settings. To establish a relationship between Evusheld for pre-exposure prophylaxis and the morbidity or mortality outcomes of COVID-19 infection, large prospective studies will be required. 

## Conclusions

Tixagevimab and cilgavimab, available as Evusheld in Singapore, were safe to use in HD patients, with no adverse events noted. It was effective in reducing COVID-19 infection as pre-exposure prophylaxis in our predominantly vaccinated patients. It led to mild symptoms in case of COVID-19 infection with a significant reduction in hospitalization rates and intensive care unit admissions. A zero mortality rate was noted in those protected by this prophylactic treatment. HD patients are immunocompromised, with Evusheld affording high-level safety for such patients who are vulnerable to severe or life-threatening infections.
